# Conquering Neutrophils

**DOI:** 10.1371/journal.ppat.1005682

**Published:** 2016-07-28

**Authors:** Simon Döhrmann, Jason N. Cole, Victor Nizet

**Affiliations:** 1 Department of Pediatrics, Division of Host-Microbe Systems and Therapeutics, UC San Diego, La Jolla, California, United States of America; 2 The School of Chemistry and Molecular Biosciences, The University of Queensland, St Lucia, Queensland, Australia; 3 Skaggs School of Pharmacy and Pharmaceutical Sciences, UC San Diego, La Jolla, California, United States of America; The University of North Carolina at Chapel Hill, UNITED STATES

## Introduction

Neutrophils are the most abundant innate immune cells, making up 50%–70% of all leukocytes. Neutrophils are the “first responders” of host defense, preventing infections by deploying sophisticated antimicrobial strategies acting in concert. As neutrophils are short-living cells, they are continuously produced and released from the bone marrow in abundance (>10^11^ per day). Circulating neutrophils are terminally differentiated, fully equipped with pre-stored antimicrobial molecules [1], and also contribute to shaping adaptive immune responses, as reviewed recently [2].

Neutrophils present challenges and limitations to experimentation, as they are short-lived, non-dividing, and genetically non-modifiable. Furthermore, no adequate cell lines exist that fully recapitulate the cellular and physiological functions of neutrophils, and murine neutrophils differ in number and (re)activity from their human counterparts. On the plus side, neutrophils can be relatively easily and quickly purified in large quantities from the blood of healthy human volunteers.

In this article, we discuss the suite of mechanisms employed by neutrophils to clear bacterial infections and the corresponding counterattack mounted by bacterial pathogens. Focusing primarily on the host response, we illustrate how a single human-specific pathogen, *Streptococcus pyogenes* (group A *Streptococcus* [GAS]), has developed an impressive range of strategies to thwart neutrophil clearance [3]. This capacity correlates to an estimated 700 million infections and 150,000 deaths annually from GAS disease, a “top 10” cause of infection-related mortality worldwide [4].

## Evidence for the Essential Role of Neutrophils in Fighting Infection

Neutrophils are potent killers of invading pathogens and rapid responders, as they migrate in large quantities to sites of infection initiated by bacteria, fungi, or parasites. The essential role of neutrophils in host defense is illustrated by genetic disorders of neutrophil function such as chronic granulomatous disease (CGD), characterized by reduced nicotinamide adenine dinucleotide phosphate (NADPH) oxidase activity and reactive oxygen species (ROS) production, or leukocyte adhesion deficiency (LAD), characterized by mutations in β2 integrin/CD18 and poor neutrophil chemotaxis, in which patients suffer recurrent infections or the high risk of invasive bacterial and fungal infections in cancer patients with chemotherapy-induced neutropenia. In mice, antibody-depletion of neutrophils is temporary, as low neutrophil numbers trigger a feedback loop to increase granulopoiesis, highlighting adaptive mechanisms in place to support the crucial defense role of these specialized leukocytes.

## GAS: A Model Invasive Human Bacterial Pathogen

Healthy individuals are at low risk for invasive bacterial infections. Yet, a few notable human pathogens are able to produce serious disease even in previously healthy children and adults. The ability of GAS to resist intact host defenses bespeaks what might be called an “innate immunity to our innate immunity.” The cause of several hundred million self-limited mucosal infections (e.g., “strep throat”) worldwide each year, GAS is also the etiologic agent of potentially life-threatening invasive infections such as necrotizing fasciitis (“flesh-eating disease”) and toxic shock syndrome. Recurrent GAS infections may trigger autoimmune diseases such as post-*streptococcal* glomerulonephritis and rheumatic heart disease [5]. GAS can be genetically manipulated and is virulent in small animal models of skin, lung, or bloodstream infections. These features allow researchers to generate and test isogenic GAS mutants to ascertain how individual virulence factors contribute to host innate immune evasion. In this article, we highlight the indispensable role of neutrophils for prevention and control of bacterial infections and how the notorious GAS pathogen subverts key neutrophil antibacterial functions to promote survival and systemic spread.

## Antibacterial Arsenal Deployed by Neutrophils and Disarming by GAS

**Fig 1:**
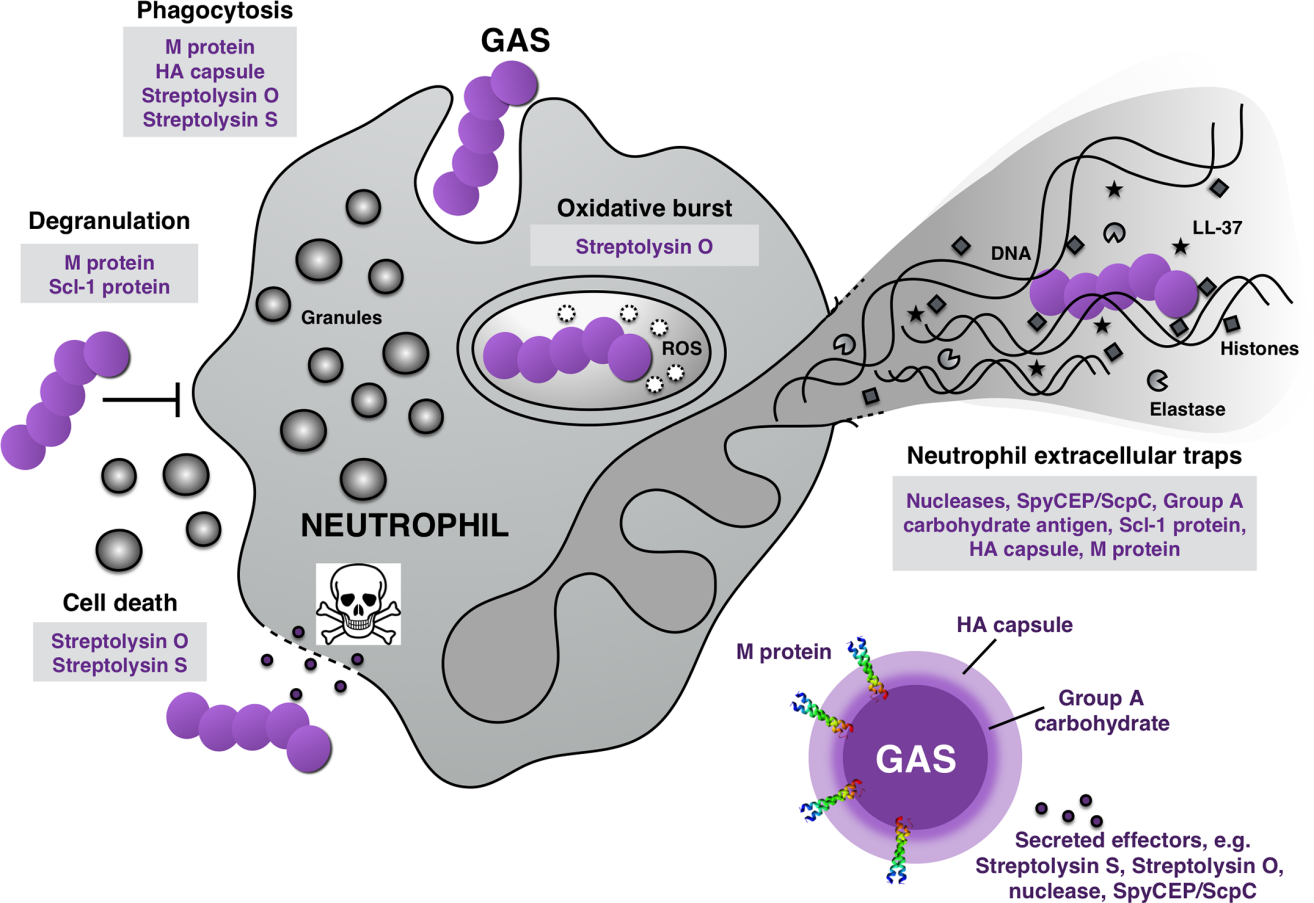
Direct anti-microbial mechanisms from neutrophils and the GAS counterattack. Neutrophils are equipped with multiple anti-infective strategies including the bacterial uptake (phagocytosis), the phagolysosomal degradation of bacteria via reactive oxygen species (oxidative burst), the release of antimicrobial molecules (degranulation), and the formation of a web-like structure composed of chromatin, histones, and antimicrobials (neutrophil extracellular traps [NETs]). GAS is equipped with a magnitude of neutrophil resistance factors (grey boxes) allowing the pathogen to uniquely counteract each anti-bacterial strategy of neutrophils.

Neutrophils are the most predominant and first innate immune cells arriving at the site of bacterial inoculation, where they exert diverse antimicrobial activities to prevent pathogen dissemination to normally sterile sites. To promote its own survival within the host, GAS has evolved an array of specific mechanisms to thwart neutrophil recruitment, phagocytosis, oxidative burst, degranulation, and neutrophil extracellular traps (NETs), summarized in Fig 1 and Table 1 and described individually below. This review focuses on GAS evasion mechanisms to neutrophil killing, but an extension of certain evasion strategies can be envisioned to apply to other innate immune cell types that are present at, or migrate toward, the site of infection.

**Table 1:** Neutrophil anti-bacterial functions subverted by GAS. GAS produces a large suite of virulence factors to counteract specific neutrophil clearance mechanisms during the pathogenesis of invasive infection.

Neutrophil function inhibited	Virulence factor	Gene	Function	Ref.
**Chemotactic recruitment**	*Streptococcus pyogenes* cell envelope protease (SpyCEP/ScpC)	*cepA*	Surface-associated serine protease that impairs neutrophil recruitment to the infection site by degrading chemokine IL-8.	[7–9]
	*Streptococcal* serine esterase (SsE)	*sse*	Secreted esterase that impairs neutrophil recruitment by inactivation of the platelet-activation factor.	[10]
	*Streptococcal* C5A peptidase A	*scpA*	C5a-peptidase that reduces complement-mediated neutrophil recruitment.	[11]
**Phagocytosis**	M protein	*emm*	Surface protein that binds complement inhibitory proteins to prevent complement deposition and phagocytosis.	[5]
	Hyaluronan (HA) capsule	*hasA*	Inhibits binding of antibodies and complement to the GAS cell surface to enhance resistance to opsonophagocytosis via molecular mimicry.	[5]
	Streptolysin S (SLS)	*sagA*	Direct cytotoxicity, inflammatory activation, and inhibition of neutrophil phagocytosis.	[14]
	Streptolysin O (SLO)	*slo*	Disrupts the integrity of host cell membranes, inducing rapid caspase-dependent apoptosis in neutrophils.	[15]
**Oxidative burst**	Streptolysin O (SLO)	*slo*	Rapid suppression of oxidative burst.	[19]
**Degranulation**	M protein	*emm*	Stimulates MPO release from neutrophils and inhibits azurophilic granule fusion with the phagosome to promote GAS intraphagosomal survival.	[21,22]
	Streptococcal collagen-like surface (Scl-1) protein	*scl1*	Inhibits the release of MPO to promote bacterial survival.	[23]
	Streptodornase 1 (Sda1)/*strepto-coccal* nuclease A (SpnA)	*sda1/spnA*	Nuclease Sda1/SpnA releases GAS entrapped in NETs by degrading the DNA backbone of NETs.	[17,31]
**NETs**	Streptococcal collagen-like surface (Scl-1) protein	*scl1*	Promotes survival within NETs by resistance to LL-37.	[23]
	Hyaluronan (HA) capsule	*hasA*	Surface trapping of LL-37 by GAS capsule prevents antimicrobial activity and engaging of inhibitory Siglec-9 by capsule impairs NET formation.	[32,33]
	M protein	*emm*	LL-37 binding by M protein prevents antimicrobial action of LL-37.	[34]
	Group A carbohydrate antigen	*gacI*	The *N*-acetyl glucosamine side chain of the group A carbohydrate cell wall polysaccharide impedes LL-37 access to the GAS cell membrane.	[35]
	SpyCEP/ScpC	*cepA*	Degradation of IL-8 impairs NET formation.	[7]

### Recruitment

Neutrophils are recruited from the blood to tissue sites of infection through a multistep cascade known as extravasation. Resident epithelial cells and macrophages at the infection site release cytokines such as interleukin-1β (IL-1β), IL-8, and tumor necrosis factor-α (TNF-α) to induce the expression of P-, E-, and L-selectins on the luminal surface of endothelial cells [6]. Circulating neutrophils attracted by the chemokines bind to the induced selectins via β-integrins, and these low-affinity interactions decelerate the neutrophil and allow it to roll along the inner surface of the blood vessel. LAD patients with β-integrin or selectin ligand deficiencies exhibit poor neutrophil chemotaxis and are more prone to recurrent bacterial infections, highlighting the crucial role of efficient granulocyte migration to the infection site. Upon penetrating the basement membrane, neutrophils migrate through interstitial space along a local chemotactic gradient. To recruit additional neutrophils, macrophages, and other immune cells to the infection site, activated neutrophils release IL-1β to stimulate the production of IL-8 by epithelial and endothelial cells in a positive feedback loop. A GAS surface-associated serine protease, SpyCEP (also designated ScpC), cleaves human IL-8 to suppress chemokine-mediated neutrophil recruitment [7]. SpyCEP enhances GAS resistance to neutrophil killing and is required for full virulence in a mouse model of systemic GAS infection [8,9]. Similarly, neutrophil recruitment is impaired via streptococcal esterase (Sse) and streptococcal C5a peptidase A (ScpA) by inactivation of the chemotactic platelet-activating factor that also contributes to bacterial virulence in vivo [10] or by cleavage of the complement factor C5a [11]. Degradation of chemotactic factors is thus a key neutrophil evasion strategy contributing to GAS pathogenesis.

### Phagocytosis

Phagocytosis is a specific form of endocytosis wherein phagocytic immune cells such as neutrophils and macrophages rapidly engulf pathogens by an actin-myosin contractile system to form a defined vacuole known as the phagosome. The phagosome subsequently fuses with a lysosome to form the phagolysosome, which contains proteolytic enzymes (e.g., lysozyme), antimicrobials (e.g., defensins, lactoferrin, LL-37), and highly toxic ROS generated by NADPH oxidase and myeloperoxidase (MPO) capable of destroying internalized pathogens [6]. Phagocytosis is activated through the binding of microbe-associated molecular patterns (MAMPs) to surface expressed receptors (e.g., Toll-like receptors) or the deposition of opsonins such as complement and antibodies on the pathogen’s surface to engage opsonic receptors on the phagocyte (e.g., FcγR and C-type lectin receptors). GAS has evolved several strategies to inhibit this process. The surface-anchored M protein inhibits phagocytosis by recruiting inhibitory complement factors (e.g., protein H, C4-binding protein), subverting antibody function through non-immune binding of the Fc domain or sequestering fibrinogen to interfere sterically with complement and antibody interactions [12]. Furthermore, the GAS hyaluronan (HA) capsule is a molecular mimic of the common host glycosaminoglycan and, therefore, provides a non-immunogenic “cloak” for the bacterium to hide surface opsonic targets from immune detection [13].

GAS pore-forming cytolytic toxins streptolysin S (SLS) [14] and streptolysin O (SLO) [15] promote resistance to phagocytosis by triggering accelerated lysis or apoptosis of immune cells, including macrophages and neutrophils. During the transition to invasive infection, mutations within the GAS control of virulence regulatory sensor kinase (*covRS*) two-component regulon are selected and result in the up-regulation of several neutrophil resistance factors, including the HA capsule and SLO, thereby, increasing resistance to neutrophil phagocytosis and killing [16,17]. Though applicable to multiple host cell types, the voiding intracellular killing via induction of accelerated cell death is of particular importance in resistance to short-lived neutrophils present in abundance during the acute stages of infection.

### Oxidative burst

Upon phagocytosis of bacteria, neutrophils and macrophages produce an oxidative (respiratory) burst resulting in the rapid release of highly bactericidal ROS, including superoxide anion, hydrogen peroxide, and hydroxyl radicals. ROS damage DNA—proteins and enzymes to which most bacteria are highly susceptible. SLO suppresses the generation of ROS independent of cytotoxicity [18]. In addition, GAS deploys a number of strategies to survive phagocyte-induced oxidative stress. GAS produces a superoxide dismutase (SodA) to enzymatically detoxify superoxide generated by neutrophils upon encounter into hydrogen peroxide. GAS harbors multiple peroxidases that subsequently decompose hydrogen peroxide [19]. Other more indirect strategies include the repair of protein or DNA damage and metal ion sequestration [19]. These evasion strategies employed by GAS provide defense against all ROS-producing cells but are especially critical to resist neutrophils, which generate the most rapid and intense oxidative burst.

### Degranulation

Degranulation is a process used to kill invading pathogens that involves the release of proteinases, e.g., neutrophil elastase (NE), MPO, and antimicrobial peptides by activated myeloid cells. Neutrophils are “pre-packed” with multiple types of granules that fuse with phagocytic vacuoles to facilitate pathogen destruction [20]. Granules also help to initiate an inflammatory response and contain alkaline phosphatase, lactoferrin, lysozyme, and NADPH oxidase [20]. The surface-anchored GAS M protein inhibits the fusion of granules with the phagosome to circumvent the host innate response and promote intraphagosomal GAS survival [21] while simultaneously triggering the extracellular release of granules potentially causing host tissue damage [22]. The surface protein streptococcal collagen-like 1 (Scl-1) protein also reduces the release of MPO, increasing bacterial neutrophil resistance [23]. In addition to neutrophils, other myeloid cell types, including mast cells and eosinophils, can release preformed granular content to kill pathogens, but it has yet to be investigated whether the GAS suppressive mechanisms facilitate immune evasion against these other cell types.

### NETs

A parallel anti-infective strategy of neutrophils involves a unique form of cell death termed NETosis [24,25]. While the full mechanistic basis of NETosis is still being elucidated, multiple neutrophil components have been shown to contribute, including NE, MPO, ROS, and peptidylarginine deiminase 4 (PAD4). Established NET-inducing stimuli include microbial factors, host immune mediators, and pharmacological agents [26]. NETs consist of extruded chromatin along with histones, allowing them to trap and kill bacterial pathogens extracellularly [27]; localized entrapment of microbes also prevents systemic dissemination [28]. NETs are highly decorated with antimicrobial molecules such as histones, LL-37, and DNA [27,29].

Several bacterial pathogens including GAS have evolved sophisticated mechanisms to suppress, escape, and/or resist NETs. One highly conserved anti-NET factor among bacteria is the expression of nucleases to degrade the DNA backbone of NETs. GAS produces nucleases that promote GAS escape from NETs, resulting in enhanced bacterial survival [17,30,31]. GAS can also suppress NET production by degrading the neutrophil stimulatory chemokine IL-8 with peptidase SpyCEP [7] or HA capsule engagement of the inhibitory neutrophil receptor Siglec-9 [32]. Other GAS resistance factors contribute to GAS resistance to antimicrobial components within the NETs by counteracting cationic peptides, including M1 protein, Scl-1 protein, and the GlcNAc side chain of the group A carbohydrate cell wall antigen [23,33–36]. These lines of experimental evidence underscore the importance of NETs in innate immunity. A similar phenomenon has been described for mast cells in innate immune defense [37], and it is likely that the GAS immune defense strategy extends to these specialized leukocytes as well. The formation of NETs represents a conserved and robust response to a large number of pathogens and has been demonstrated in vitro and in vivo.

## Future Perspectives on Boosting Neutrophil Antimicrobial Activity

Humans with dysfunctional neutrophils or low neutrophil numbers are at high risk for invasive and recurrent (bacterial) infections. The medical challenge is more pressing as several important human pathogens develop multi-drug resistance, and antibiotics become increasingly less effective. Therefore, continued research to define pathways by which neutrophil function can be supported to counteract the intrinsic resistance mechanism of leading bacterial pathogens is warranted. For instance, pharmacological strategies to augment the antimicrobial functions of neutrophils in the context of acute infection have emerged as new avenues of research to attempt to overcome antibiotic resistance and neutrophil resistance strategies employed by leading pathogens such as GAS. For example, stabilization of the transcriptional regulator hypoxia-inducible factor 1 (HIF-1) enhances neutrophil energy generation, antimicrobial activities, and treatment outcomes in a mouse model [38], innate defense regulator peptides (IDRs) increase neutrophil antimicrobial peptide production and bacterial killing [39,40], and nicotinamide (vitamin B3) boosts neutrophil bactericidal activity to provide prophylactic and therapeutic activity against *Staphylococcus aureus* in vivo [41]. Furthermore, NET formation and bacterial killing are boosted in vitro and in vivo by treatment with familiar pharmacological agents such as the breast cancer drug tamoxifen [42] or cholesterol-lowering statins [43]. Indeed, the use of statins is currently in phase IV clinical trials (EudraCT number: 2012-003343-29) to enhance the antimicrobial activities of neutrophils in elderly patients with septic pneumonia [44].

Although these alternative approaches are still in preclinical or early clinical development, boosting neutrophil function during infection has the potential to provide a critical new element to the treatment of potentially life-threatening antibiotic-resistant infections by harnessing the multifaceted antimicrobial properties of these sentinel immune defense cells. In contrast to broad-spectrum antibiotics, host-directed strategies may minimize collateral effects on the human microbiome [45] and the risk for development of antibiotic resistance. New treatment options are desperately needed in face of the continual emergence of multi-drug–resistant bacterial pathogens and the paucity of new candidates in the antibiotic development pipeline.
